# Advanced HIV disease and health related suffering- exploring the unmet need of palliative care

**DOI:** 10.1016/S2352-3018(22)00295-8

**Published:** 2022-11-22

**Authors:** Ajay Rangaraj, Stephen Connor, Richard Harding, Clarice Pinto, Lastone Chitembo, Nathan Ford

**Affiliations:** 1 World Health Organization, Geneva; 2 Worldwide Hospice Palliative Care Alliance, UK; 3 Cicely Saunders Institute of Palliative Care, Policy & Rehabilitation

## Abstract

With over 38 million people living with HIV worldwide, the scale-up of antiretroviral therapy (ART) ensures nearly 28 million receive regular treatment. However, still a significant number of deaths occur every year from AIDS-related complications, approximately 680,000 in 2021. 7 million people out of an estimated 56.8 million globally in need of palliative care can access services. Providing palliative care services can help alleviate health related suffering, reduce pain, other symptoms and improve well-being. This paper discusses the unrealised potential of palliative care in individuals with advanced HIV disease. Key areas of health worker training include appropriate sensitisation, training in palliative care and effective communication. Offering advance care planning is of vital importance, impacting both the individual and their family. Integration of palliative care into HIV programs is needed to address health related suffering, particularly for AHD, and accelerate global efforts for ending AIDS related deaths by 2030.

## Introduction

As of June 2021, it is estimated that there are approximately 38 million people living with HIV, and an ever-increasing proportion that are receiving antiretroviral therapy (ART), with currently over 28 million people receiving ART(1, 2). The rapid scale up of access to treatment - in particular following the policy to Treat-All introduced in 2015(3) – has greatly improved survival as well as quality of life in people living with HIV (PLHIV). However, there were some 680,000 AIDS related deaths in 2020 with coinfections such as tuberculosis, cryptococcal meningitis and severe bacterial infections as leading causes(1).

Individuals with advanced HIV disease are at greatly increased risk of mortality from a weakened immune system and chronic systemic inflammation. Mortality in this group occurs within the first few weeks after presentation to care and many of those deaths occurred at home – most likely due not from a conscious choice, but an inability to access health services in time for live saving interventions to be offered. (4) Many individuals with AHD are very likely to be hospitalised multiple times through the course of their lifespan and have relatively poor outcomes following discharge from hospital(5).

The World Health Organization(WHO) defines palliative care as an approach to improve the quality of life of patients and their families who are facing problems associated with life-threatening illness(6). It is estimated the 78% of adults and 98% of children in need of palliative care reside in low and middle income countries (LMICs), with only a small proportion receiving it(6, 7). The latest WHO consolidated guidelines on HIV now incorporates a new section on palliative care as part of HIV services. Globally it is estimated that palliative care is needed in up to 60% of deaths that occur. Is it estimated that only 7 million out of an 56.8 million people in need of palliative care actually receive it – which highlights an enormous unmet need(6, 8).

There is evidence that timely intervention with palliative care can have a positive impact among those admitted to hospital and on quality of life for those living with HIV(9). This paper will summarize the role that palliative care service integration with national HIV programmes can play in achieving the UN Sustainable Development Goal of ending AIDS related deaths by 2030(10).

## Mortality and morbidity from advanced HIV disease

In 2017, WHO recommended the use of a package of care to manage advanced HIV disease, primarily targeted at outpatient-based HIV programming(11). This guidance aims to help programs manage individuals who are not seriously ill. Among individuals requiring hospitalisation, those living with HIV are responsible for a substantial proportion of mortality in LMICs, with reports as high as 50% in some settings(12). Further, the clinical course in these individuals is often complicated – having multiple infections(13) simultaneously, post-infectious sequelae, and reduced functional status(14) in some instances. Individuals with AHD, often present with a WHO clinical Stage 3 or 4 illnesses, which are tabulated on Page 1 of the web appendix A recent systematic review estimated that post- discharge mortality among PLHIV was 15%, and a readmission rate of nearly 19%(5). The post discharge mortality rate was noted to be higher in the African continent (23%) when compared to the USA (7%). The pooled readmission and mortality was found to be approximately 32%(5). Several factors were found to contribute to negative outcomes, including a lack of linkage to care following discharge, discharge against medical advice, low CD4 cell count at admission and longer length of stay. Another concerning trend is that an increasing proportion of individuals with AHD enrolling in care, disengage from care for a variety of reasons, are re-entering care following disengagement, and are more likely to have advanced HIV disease(15). This has significant implications both in terms of choice of antiretroviral regimen due to concerns of drug resistance (as these individuals will have interrupted their treatment), as well as offering timely interventions for potentially life-threatening co-infections stemming from poor immune status.

Many individuals, including those who are aware of their HIV status prior to hospital admission, are not appropriately linked to outpatient care following discharge(16). Individuals on ART now live longer lives and therefore are at greater risk of noncommunicable disease comorbidities as they age, made worse by chronic inflammation due to HIV itself. People living with HIV consistently have higher cardiovascular risks (17, 18). There is also an increased risk for PLHIVs to experience poor mental health, which in turn will affect health seeking behaviour, adherence, and quality of life; this is further worsened by health, social inequities and finally, social stigmatisation(19–21). These issues are substantially worse in individuals who belong to key populations(22, 23). Unrelieved health-related suffering including anxiety and depression(6, 7) is highly prevalent in people living with HIV, including among those on ART(6, 7). In addition, co-infections, comorbidities, side effects or adverse effects from medications are particularly common in people with advanced disease(24) which further exacerbates health related suffering.

There is a need to engage patients and their families in decision making for their healthcare, with evidence demonstrating positive outcomes when there is good engagement(25). Most PHIV worldwide are supported through outpatient services, leaving little room to discuss palliative care needs due to competing programmatic priorities. There is a need for palliative care to be considered as an integral part of the HIV care continuum(26).

These challenges combine to manifest as unresolved health-related suffering as well as clinical uncertainty, which has implications for PLHIV when seriously ill and enrolled in care. It should not be forgotten that unresolved suffering has a negative impact on outcomes relating to ART in terms of morbidity and mortality(27). Thus, a framework is required to account for the various challenges that individuals with AHD face, including children living with HIV who are often underserved in the HIV response, retaining a person-centred approach within a public health framework(28).

## Unrealised potential and unmet need of palliative care

A report released in 2020 by WHO and the Worldwide Hospice Palliative Care Alliance estimated that globally over a quarter (28%) of the need for palliative care are among people living with HIV and this need rises to 33% in LMICS(6). WHO provides a comprehensive definition of palliative care, which aims to relieve suffering through “early identification, correct assessment and treatment of pain and other problems, whether physical, psychosocial or spiritual”, and stresses that palliative care is not limited to any single care setting. It can be divided into three major categories, namely acute care, long-term care and home care, which span palliative services in a hospital, nursing/residential home and at general practitioner or community nursing teams(6). Despite the clear benefits of scale-up of ART globally – there are still many preventable deaths observed among people living with HIV and in addition to an unmet need for management of serious health related suffering and end of life care. A clear understanding of where people living with HIV die would help inform where interventions should be delivered to facilitate individuals and their families, and improve quality of services(29).

Availability of palliative care in many high-income settings as well as in some low-income settings has demonstrated great effectiveness in improving the quality of life in people with HIV, with appropriate pain management reducing length of hospital stay as well preventing readmissions and ensuring that those admitted with a terminal illness are given a choice to continue a hospital stay or return home(9). The Global Atlas of palliative care reports that in the WHO AFRO region alone, 78% of individuals in need of palliative care are among people living with HIV, but availability of services is heavily concentrated in European countries(6).

A systematic review of 11 countries showed home deaths were the least common place for an individual living with HIV to die(29). Nevertheless, some studies report a high proportion of people dying at home(4). Among the 11 countries included in the review, only Mexico reported a percentage of 26.3% of HIV related deaths occurred at home. It is expected that in countries where access to health facilities is impaired or is challenging – home deaths will constitute a larger proportion of place of death among PLHIVs, as reported in the REALITY trial, with a significant proportion dying within 8-10 weeks after initiation of ART(4). The key take-away of these seemingly contradictory findings is that there is an absence of choice for the patient to decide where they would prefer to die in both these instances – whether occurring in settings with good access to health services – or in its absence, as witnessed in the REALITY trial.

A scoping review done in 2016 identified several countries that are gradually scaling up government-endorsed palliative care systems, including Malawi, Zimbabwe, Rwanda, Eswatini, Tanzania and Zimbabwe(30). An emphasis on palliative care is largely absent in many clinical practice guidelines, and even at country level(6, 7). Almost a quarter of countries worldwide (47 countries), have no known palliative care activity; 33% of countries provide isolated or targeted palliative care and just 11% of countries worldwide provide generalised palliative services. At the current time, just over 5.7% of the world’s population received palliative care that is standardised and consistently available, and is focussed for the most part on cancer related care (6).

The unmet demand for palliative care is an urgent need, with some modelling projections suggesting that the burden of health-related suffering will have doubled by the year 2060, with a projected increase of 155% between 2016 to 2060 in low- and middle-income countries(31). Recent estimates from China(32) suggest that 53% of PLHIVs had at least one symptom that was unmanaged; another study from Zambia reporting similar numbers of unmet pain symptoms(33). Fatigue is known to be a frequent symptom in PLHIVs and in chronic disease and this has far reaching implications on the quality of the life-course for PLHIVs, including a negative effect on ART adherence(9). Findings such as frequent reports of unresolved pain and poor mental health are repeatedly(34, 35) observed in PLHIVs in a range of qualitative studies, semi-structured interviews and through quality-of-life assessments. Declines in wellbeing were noted in both HIV positive and negative populations where there was physical decline associated with chronic illness based on findings from two separate tools designed to measure wellbeing, physical function – which highlights a need for physical rehabilitation as well(36).

One approach to better understand the range of symptoms PLHIV suffer from has focussed on examining ‘symptom clusters’, based on the assumption that certain symptoms tend to occur together. A systematic review by Zhu, et al showed that there were five most common ‘symptom clusters’, the first included sadness/depression/loss of interest/nervous/anxiety/worry; the second was difficulty sleeping/sexual problems, fatigue/loss of energy; the third being fever/chills/nausea/vomiting and loss of appetite; then numbness, muscle aches, joint pain; and finally dizziness and headache. They also noted that physical symptoms such as fever and chills were usually reported alongside other systemic symptoms such as gastric pain or discomfort, while other symptoms such as loss of appetite could also occur independently.

Importantly, the study also highlighted that there is a broad range of tools used to measure and evaluate symptoms, with little uniformity across methods of evaluation(37). In summary, the symptoms of health-related suffering are diverse, frequent, and are not dealt with adequately – and healthcare workers, without prior sensitisation to palliative care needs are likely to overlook symptoms and perpetuate health-related suffering.

Other limitations of current practices include a lack of clarity on options for home-based care following discharge and choice on place of death(38). Home-based palliative care provision impacts families and are often challenging to manage in the absence of knowledge or social support, whether for adults, adolescents or children(39). The relationships between an individual and their families can also be difficult – with stigma and discrimination also arising within families – such as separation of belongings, eating utensils, separation from children in the household, eviction from home, which are all from a lack of health literacy in relation to HIV disease(40). A study from Malawi describes a range of perceived needs for home-based care, including the need for providing physical care by primary caregivers- such as assistance with bathing, assistance with mobility or changing positions in bed and also wound care in PLHIV(41). This is further worsened if an individuals’ primary caregivers lack the knowledge on the care that is needed, which could be the case in some settings(42).

Other limitations of current practices include a lack of clarity on options for home-based care following discharge and choice on place of death(38). Another report from Rwanda indicated that 50% of professionals had not received any form of palliative care training(43).

A study that documented lived experiences of PLHIVs in India highlighted several concerns of individuals and their families, describing a fear of death, a need for better transparency regarding a poor prognosis, and a general lack of awareness among both patients, their families and health care workers offering hospitalised care(38). In many settings, there is a gap in provision of adequate training in palliative care to general practitioners particularly for cancer care(44) as well as part of training in post-graduate medical curricula(45).

A systematic review from 2015 identified that existing literature on palliative care in SAARC (South Asian Association of Regional Cooperation) countries did not match the estimated size of population in need, also highlighting there were no papers found from Sri Lanka, Bhutan, Maldives, or Bangladesh -which highlights the scope and scale of challenges faced by people living with HIV – as little information is available for the general population – and even less in the case of HIV(46).

## What needs to be done?

WHO provides guidance on how to scale up palliative care in a variety of settings, with different levels of expertise, training needs as available resources(47). Although limited cost-effectiveness studies exist on the subject, the timely offer of palliative care reduced hospital readmissions and shortens length of stay both of which will contribute to reduced costs to the individual and the health system(9). Many national HIV programmes have moved towards community-based models of care applying differentiated service delivery models to PLHIVs established on ART, to respond and adapt to specific needs of certain communities, geographic context, and health system structures to provide ART and increase retention to treatment. There is a trend towards de-medicalisation of HIV care, including self-care, with the goal of simplifying and sustaining access to services, moving away from a one-size-fits all approach to a person-centred approach that puts individuals in the position to decide which HIV service delivery interventions best fit their needs(48).

Individuals with AHD need greater medical attention, access to health facilities, and often require hospitalisation for a variety of needs,(24) including timely delivery of live-saving antimicrobials. The adoption of a comprehensive palliative care framework accompanied with an appropriate service delivery model will facilitate smoother transitions in and out of hospital-based care and outpatient care, reduce the need for readmissions and ultimately ensure comfort and relief from suffering of the individual and their families and provides a choice, in the event they face an overwhelming illness, as to where they would prefer to die. Better understanding of place and causes of death in different settings(49, 50) would help inform the development of a suitable palliative care model, as well as define workforce requirements, training, and access to pain medications.

As in most resource- limited settings, costs are a major consideration- but costs as low as 75-175 USD were demonstrated in India for provision of a basic set of services for palliative, and this can be reduced further, to 50USD(7), once economies of scale are achieved. The WHO Essential list of Medicines includes a number of key pain alleviation medications, including Morphine;(51) however, morphine, being an opioid, is heavily regulated, is seldom available in oral formulations nor available widely(52). A systematic review of costs accrued at the end-of-life in LMICs found that along with several other diseases, a diagnosis of AIDS had potential for catastrophic expenses and out of pocket payments in settings with weaker health infrastructure(53). Inaction in this regard thus has a profoundly negative impact on the patient, their families and for health systems.

The first step towards a sector-wide response is to understand the needs of individuals with AHD as well as how they would choose to best receive care, i.e., a palliative care framework should be context-driven(54). Further, involving families of the individual when appropriate is also important, in terms of improving health literacy and offering social support. Involving families for advance care planning is also critical, and appropriate involvement results in improved understanding of the individual’s treatment-related preferences and also ensures they are able to document their advance directives(55, 56). The next step would be to evaluate the opportunities for integrating care within HIV program structures against a minimum requirement for offering palliative care.

Table 1 describes in the first column the typical workforce of an HIV clinic(57), describing their roles and accounting for various task-sharing activities. The following three columns show the pre-requisite training in order to offer palliative care at facility- and community-level, as well as home-based care as outlined by the WHO document on quality health services and palliative care offers practical approaches and guidance on allotment resources to support policy, strategy, and practice to establish services in countries with limited resources(47) at all levels of healthcare.

Thus, to offer a basic package of palliative care services, major additions to the workforce are unlikely to be necessary, as existing staff may be trained to function at the required level of care (hospital, community or home-based). Advance care planning or end-of-life planning should be offered to those who are terminally ill, to enable the provision of choice for patients and their families. Special attention is needed for vulnerable groups, who are often underserved in term of advance care planning(58). Specialised care at a facility is notably more complex, requiring better infrastructure and an on-site palliative care physician. However, basic training of health cadres immediately enables them to offer care at community-level and in homes. Investment by national programs in trainings would represent a vital step towards integration of palliative care with HIV care.

**Figure 1:**
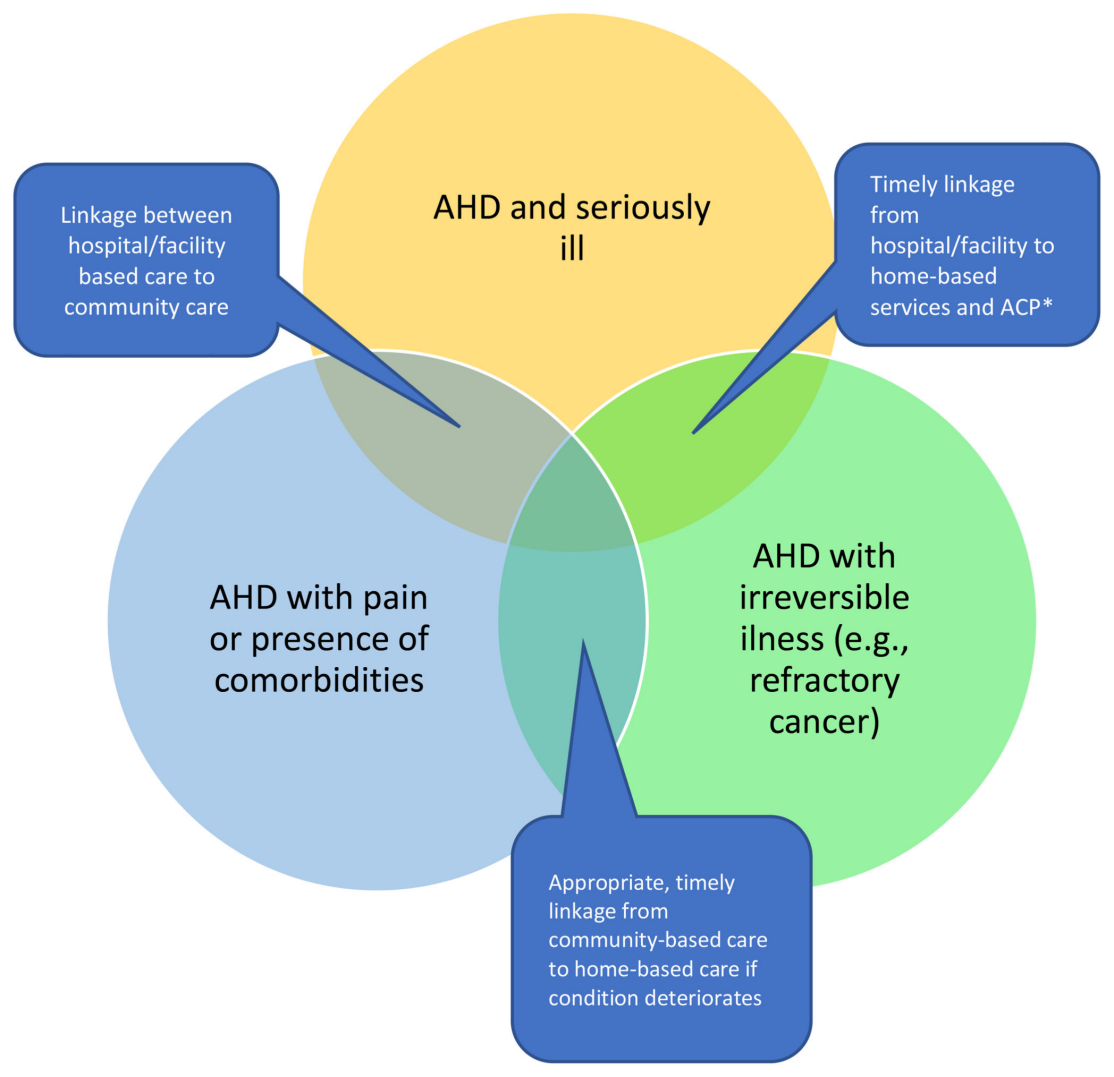
Visualisation of palliative care continuum in individuals with advanced HIV disease. Home-based care models may require advance care planning where appropriate and necessary *ACP- advance care planning

Figure 1 illustrates the approach for integrating a palliative care model into routine HIV care, utilising existing frameworks(47) of care, distributed by physical state and functional capacity of individuals with AHD in the HIV care continuum – it is critical that linkages are strengthened between hospitals, HIV clinics and community-based care in order to ensure that individuals with AHD discharged from hospital are adequately counselled, offered social support, have their symptoms managed and are linked to care to continue receiving their ART. Integration of palliative care services will allow for smooth transitions between inpatient and outpatient care as part of the HIV care continuum.

In terms of outcomes, there exists a clear link between outcomes for individuals in need of palliative care and outcomes – a systematic review that included 43 clinical trials, with over 12,000 patients found that offering palliative care to those in need resulted in statistically and clinically significant improvements in quality-of-life measures and symptom burden(27). Another review describes that a number of interventions that could be implemented in low-resource settings exist, but there is a lack of high quality evidence to document outcomes in LMIC settings, reflecting a clear need in terms of research focus(59). Additionally, there have been demonstration of effectiveness of implementing palliative care in outpatient HIV programs, which demonstrated statistically significant reduction in pain and symptoms(9).

There is also evidence to suggest that palliative care may also help improve resistance to stigma and discrimination, as previously discussed(60). The timing of offering palliative care is also an important consideration, particularly in some sub-populations, such as in adolescents, where early palliative care and advanced care planning demonstrated a benefit in relation to symptom burden.

The role of religious or traditional healers or counsellors, complementary or alternative medicine could be important. A Cochrane systematic review concluded that the benefits of spiritual and religious interventions are inconclusive, as none of the studies in the review examined benefits to the recipient of care(61). Another review on complementary and alternative medicine in palliative care found short term improvements in symptoms(62). Preferences for seeking care may depend on the cultural context and extent of involvement of healers and for receiving alternative or complementary medicine. Some insights from a qualitative study conducted in South Africa suggested that there was a desire for and understanding of local spiritual and cultural customs of dying, death, bereavement, and indigenous knowledge when providing care, particularly in rural areas. This arose from the belief that traditional healers had a more in-depth knowledge of an individual’s psychological needs and could thus complement the work of health workers offering home-based care(63). The literature on this specific issue is very limited and more research could help elicit a link between palliative care and traditional/alternative medicine in terms of outcomes.

## Conclusions

It is clear there are three non-parallel workstreams that ideally should be linked and integrated with one another: (1) further scale-up of antiretroviral treatment through outpatient programs (2) greater implementation of the WHO recommended advanced HIV disease package of care(24) to reduce mortality from AHD and (3), a comprehensive palliative care model for people living with HIV that will bridge the service gap between outpatient and inpatient care.

There is very limited availability of palliative care services worldwide despite the demonstration of effectiveness in terms of reducing pain, symptom management and advance care planning. The greatly reduced mortality from improved HIV testing and ART scale-up has been replaced with a growing burden of health-related suffering among PLHIV with AHD that has a significant impact on the quality of their lives, as well as their families. Future research should focus to document outcomes for individuals with AHD when palliative care is offered.

**Figure 2:**
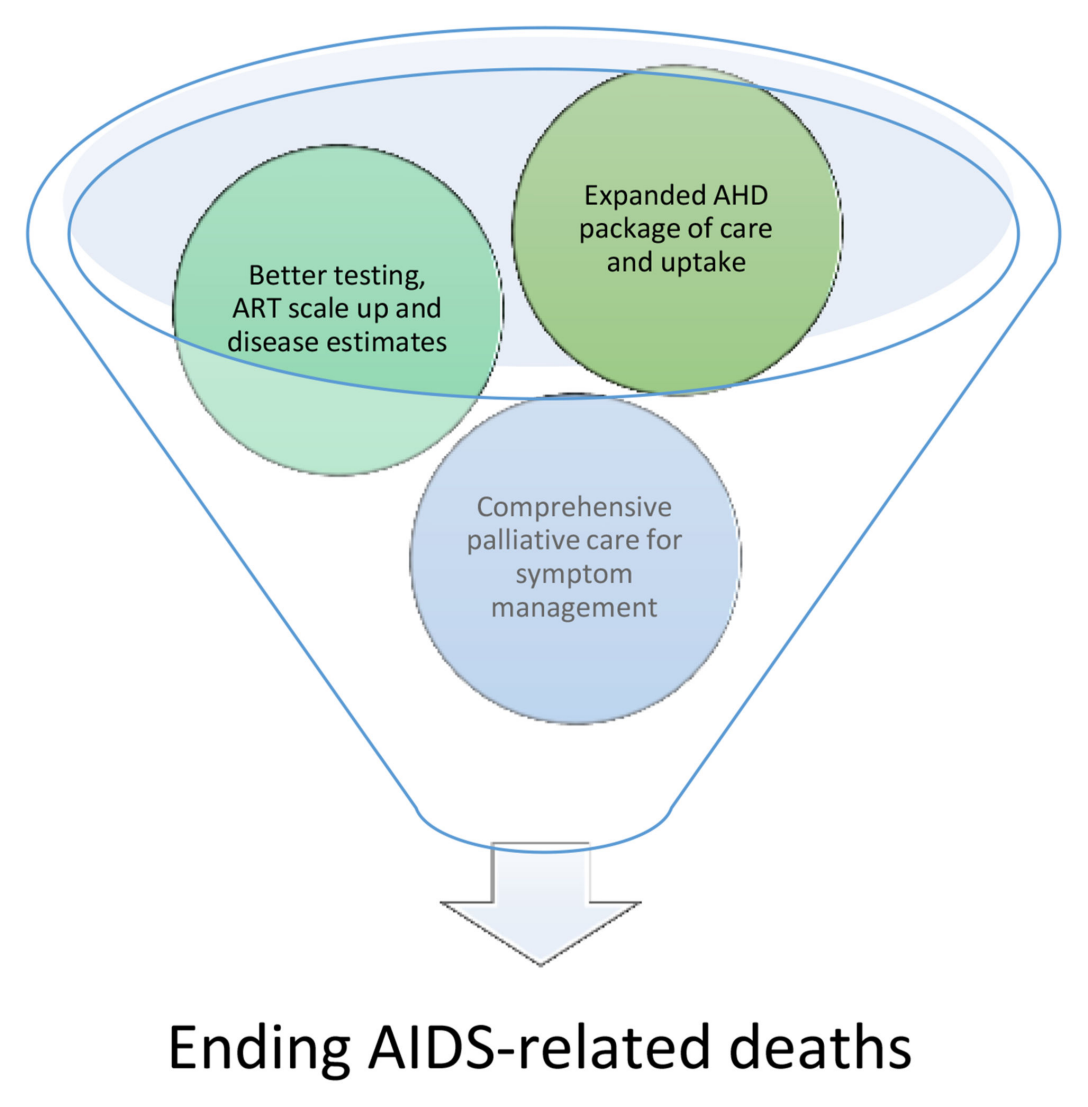
Ingredients for success in ending-AIDS related deaths by 2030

Depicted visually in Figure 2, unity of these three workstreams will strengthen the HIV care continuum, and will thus be a significant boost to achieving the target of ending AIDS related deaths by 2030(64). Finally, the growing burden of non-communicable diseases (NCDs) and its impact on morbidity and mortality among those with AHD is not to be underestimated(65)(66), and palliative care is needed urgently to provide quality health services for people living with HIV and ensure maintenance of health throughout their life-course, looking beyond the 2030 target of ending-AIDS(64).

## Figures and Tables

**Table 2:** Typical workforce in an HIV clinic and additional training needs for offering palliative care services

Typical HIV clinic workforce & duties	Additional training needs to offer palliative care services within HIV programs by healthcare level			Additional tasks to be undertaken for offering palliative care
	Facility-level*	Community-level	Home-based care	
**Medical doctor/ clinical officer**: ART prescription, monitoring, management, referrals	Training** in palliative care and communication skills. As a minimum, a course with a theoretical component and at least 10 days’ practical. Ideally, a specialist qualified in palliative care would be present	Foundation course of 3-10 days or mid-level training for a 6-week residential course	Foundation course for 3-10 days	To provide medical support services, symptom management Palliative care at hospital level – will require a physician specialised in palliative medicine
**Nurse/Midwife**: registration, ART, initiation and dispensing, adherence counselling, phlebotomy, patient tracing, medical records management	Training in palliative care and communication skills. As a minimum, a course with a theoretical component and at least 10 days’ practical. Ideally a more specialized qualification in palliative care.	6 weeks of residential training supported by auxiliary nurses with foundation training in palliative care	Foundation course for 3-10 days	To provide nurse-led supportive care, medical support in certain settings and contexts
**Counsellor**: triage, ART adherence counselling, patient tracing for reengagement, ART dispensing	Trained counsellor with orientation to special needs in palliative care.			
**Pharmacist/ Pharmacy technician**: ART dispensing	No additional training, parttime role with the team, support in dispensing pain medications/ other medications for symptom management			
**Lay health worker/volunteers (trained/untrained)**: registration, patient tracing	Based on context of their existing roles, serve as additional support to the team	*Trained volunteers:* 16 hours of theory plus 4 home visit days covering communication skills, emotional support, patient assessment, nursing care, home care, basic symptom management and reporting to a higher level *Untrained volunteers:* Sensitisation course (approx. 2 hours) on basics of palliative care, home care and communication	All Volunteers: 3-hour introductory course	Contribute to care by offering emotional support, basic nursing tasks, assistance with mobility, reporting of uncontrolled distress to higher level Untrained volunteers can provide other services relevant to palliative care: transport, food for patients and fund-raising
**Community health worker/ social workers**: patients tracing for missed appointments	Trained social worker with orientation to special needs in palliative care.	Basic course of 3-6 hours covering communication skills, emotional support, patient assessment and reporting to a higher level	3-hour introductory course or a basic course	Contribute to care by offering emotional support, basic nursing chores, assistance with mobility and reporting of uncontrolled distress to higher level

Other key members of the HIV clinic workforce include laboratory technicians and data clerks, and do not necessarily have a specific role in offering palliative care

* Psychologists or physiatrists’ may be included as part of a specialised team at facility-level or would accept referrals to cater to mental health assessments and needs

** Training in this table refers to the training schedules, curricula and frameworks mentioned in the WHO document titled “Planning and implementing palliative care services: a guide for program managers, 2016”

References for Table 2: 1. Tsui S, Denison JA, Kennedy CE, Chang LW, Koole O, Torpey K, et al. Identifying models of HIV care and treatment service delivery in Tanzania, Uganda, and Zambia using cluster analysis and Delphi survey. BMC health services research. 2017;17(1):1-11. 2. Planning and implementing palliative care services: a guide for programme managers. Geneva: World Health Organization; 2016. Report No.: 9241565411.
